# Declining Frequency of Acute Complications Associated with Tubeless Insulin Pump Use: Data from 2,911 Patients in the German/Austrian Diabetes Patienten Verlaufsdokumentation Registry

**DOI:** 10.1089/dia.2020.0675

**Published:** 2021-08-04

**Authors:** Torben Biester, Anke Schwandt, Bettina Heidtmann, Birgit Rami-Merhar, Thomas Haak, Andreas Festa, Susanne Kostow, Antonia Müller, Kirsten Mönkemöller, Thomas Danne

**Affiliations:** ^1^ AUF DER BULT Diabetes Center for Children and Adolescents, Hannover, Germany.; ^2^ Institute for Epidemiology and Medical Biometry,ZIBMT,Ulm University, Ulm, Germany.; ^3^ German Center for Diabetes Research (DZD),Neuherberg, Germany.; ^4^ Catholic Children's Hospital Wilhelmstift,Hamburg, Germany.; ^5^ Department of Pediatric and Adolescent Medicine,Medical University of Vienna, Vienna, Austria.; ^6^ Diabetes Clinic Bad Mergentheim,Bad Mergentheim,Germany.; ^7^ Landesklinikum Korneuburg Stockerau,Korneuburg Stockerau,Austria.; ^8^ Sana Klinikum Lichtenberg—Oskar-Ziethen-Krankenhaus,Berlin,Germany.; ^9^ Klinik für Stoffwechsel und Diabetes,Karlsburg,Germany.; ^10^ Kinderklinik Amsterdamerstrasse, Köln, Germany.

**Keywords:** Hypoglycemia, Diabetic ketoacidosis, CSII, DPV, Omnipod, patch pump

## Abstract

***Objective:*** To characterize patients with diabetes treated with a tubeless insulin pump (Omnipod^®^ Insulin Management System; Insulet Corp., Acton, MA), and to evaluate the frequency of acute complications with long-term use of the system.

***Methods:*** This retrospective analysis of the German/Austrian Diabetes Patienten Verlaufsdokumentation (DPV) registry included data from 3657 patients with diabetes (*n* = 3582 type 1, *n* = 25 type 2, *n* = 50 latent autoimmune diabetes in adults/other) treated with a tubeless insulin pump. Hemoglobin A1c (HbA1c) levels and frequency of diabetic ketoacidosis (DKA) and severe hypoglycemia (SH) were compared between 1 year pre- and 1 year (*n* = 2911) or up to 3 years (*n* = 1311) post-tubeless insulin pump initiation and compared with a contemporary cohort on multiple daily injections (MDI) with 3-year data (*n* = 1874).

***Results:*** Patients using tubeless insulin pump therapy had a median age of 13.7 years [interquartile range 10.8, 17.3], diabetes duration 3.7 years [1.7, 8.0], and HbA1c 7.5% [6.9, 8.2]. In patients with 3 years of follow-up data (*n* = 1311), the percentage with ≥1 episode of DKA, SH (Level 3, requiring assistance), and SH (coma) event with prior treatment was 6.3%, 5.5%, and 1.7%, respectively. After 3 years of tubeless insulin pump therapy, the frequency of DKA, SH (Level 3), and SH (coma) decreased to 2.2%, 4.1%, and 0.5%, respectively. Both DKA and SH remained significantly lower compared with MDI after adjustment in multiple regression analysis. High treatment retention rates (>90%) were observed.

***Conclusion:*** Real-world registry data document that tubeless insulin pump therapy is associated with good glycemic control and a low frequency of DKA and SH in an age group prone to acute complications.

## Introduction

Severe hypoglycemia (SH) and diabetic ketoacidosis (DKA) are acute complications of diabetes that can require treatment through emergency department visits and hospitalization, and can quickly escalate to life-threatening situations.^1–8^ These acute complications can also have a negative impact on patients' overall well-being and quality of life and contribute to diabetes-related psychological distress,^9–14^ in addition to the added burden of costs to patients, their families, and the health care system.^15–20^

Despite continued innovation in diabetes treatments and technologies, DKA and SH rates remain relatively high worldwide, with large national registry data showing estimates of 2% to 16% of patients affected per year, varying by age group, treatment modality, country, and other factors.^2–8,21–29^ Encouragingly, recent studies suggest that insulin pump use is associated with a lower proportion of subjects with acute complications than multiple daily injections (MDI) in youth with type 1 diabetes.^3–6^

A previous retrospective analysis of German/Austrian Diabetes Patienten Verlaufsdokumentation (DPV) registry data indicated that treatment with a tubeless insulin pump (Omnipod^®^ Insulin Management System; Insulet Corp., Acton, MA) may be associated with improvement in glycemic outcomes in youth with type 1 diabetes compared with treatment with MDI.^30^ The objective of this study was to characterize patients of all ages with diabetes treated with a tubeless insulin pump in the German/Austrian DPV registry and to evaluate the frequency of acute complications after tubeless insulin pump initiation, which has not previously been reported.

## Research Design and Methods

The German/Austrian/Swiss/Luxembourgian DPV registry has been described previously.^31^ In short, the DPV initiative collects data on patients with diabetes mellitus every 6 months using DPV software and the anonymized data are sent to the University of Ulm for aggregation into the database. The DPV initiative was established in 1995, approved by the University of Ulm Ethics Committee, and data collection was approved by local review boards.

We evaluated the clinical characteristics of all patients with diabetes treated with a tubeless insulin pump, as well as glycemic control and the frequency of acute complications post-tubeless insulin pump initiation compared with prior treatment. Patients with any diabetes diagnosis (type 1, type 2, and latent autoimmune diabetes in adults [LADA]/other) who initiated treatment with a tubeless insulin pump from January 1, 2013, to March 2019 were included in the summary of demographics (entire cohort).

Glycemic control and frequencies of acute complications over time were analyzed for patients who had at least 1 year of data before tubeless insulin pump initiation and at least 1 year of follow-up data available (referred to as total cohort), and for a subgroup of those patients with 3 years of follow-up data available. In addition, we compared this with MDI patients from the same centers that also had 3 years data available during this time period. For the MDI group, the “Year Prior” corresponds to the third year before last year evaluated.

### Outcome measures

The demographics, including age, hemoglobin A1c (HbA1c), insulin dose, and body mass index-standard deviation score (BMI-SDS) using the international pediatric reference data from the World Health Organization (WHO) (www.who.int/childgrowth/standards/bmi_for_age/en/), of patients using the tubeless insulin pump were summarized, both overall and stratified by age group. Glycemic control and proportion of subjects with DKA and SH were analyzed for the year before switch to tubeless insulin pump (using prior treatment modality) and at 1, 2, and 3 years post-tubeless insulin pump initiation.

DKA was defined as pH <7.3 or bicarbonate concentration <15 mmol/L.^32^ To avoid skewing of the analysis, single patients with multiple DKA events per year were counted as 1. SH was evaluated both as SH (Level 3), defined as blood glucose (BG) <70 mg/dL or <3.9 mmol/L and requiring assistance from another person to actively administer carbohydrates, glucagon, or intravenous glucose, and SH (coma), defined as loss of consciousness or occurrence of seizures.^33^ Retention rate on tubeless insulin pump therapy was also determined.

### Statistical methods

Results are presented as median (interquartile range, IQR) or mean (standard deviation) for continuous variables, and as proportions for binary variables. Kruskal–Wallis test was used for group comparisons of continuous variables. Nonparametric statistics were used because most outcome measurements were not normally distributed. Chi-squared test was used for comparison of dichotomous variables.

Multiple regression models were applied for the outcome variables HbA1c, total daily dose of insulin, and BMI SDS, and logistic regression models were applied for SH and DKA to control for differences in age, gender, and diabetes duration between treatment groups. Mathematical details of the regression models, as well as the implementation in the SAS software, are described elsewhere.^34^ Two-sided hypotheses were used throughout the analysis. A *P*-value <0.05 was considered statistically significant. The statistical analysis software package SAS, version 9.4 (SAS Institute, Carey, NC), was used for all analyses.

## Results

The German/Austrian DPV registry includes 3657 patients with diabetes using the tubeless insulin management system. Within this cohort, 2911 patients had at least 1 year of data using prior treatment and 1 year of follow-up data post-tubeless insulin pump initiation. A subgroup of 1311 patients had 3 years of follow-up data post-tubeless insulin pump initiation.

### Clinical characteristics

Clinical characteristics of the entire cohort of patients using the tubeless insulin pump as of March 2019 are summarized in Table 1. The distribution of patients by diagnosis is primarily type 1 diabetes (*n* = 3582, 98%), with a small number of patients with type 2 diabetes (*n* = 25) and LADA/other diagnoses (*n* = 50). Overall, patients were median (IQR): age 13.7 years (10.8, 17.3), diabetes duration 3.7 years (1.7, 8.0), and HbA1c 7.5% (6.9, 8.2). The majority of patients were <20 years old (*n* = 3023, 83%). Continuous glucose monitor (CGM) use was higher in the pediatric age group than in adults. The prior treatment modality for patients with type 1 diabetes was 58.5% MDI, 38.1% other pump, 3.2% tubeless insulin pump as initial therapy, and 0.3% unknown. The median duration of tubeless insulin pump use was 1.1 (0.1–2.7) years overall and comparable for the subset of patients with type 1 diabetes: 1.3 (0.1–2.3) years.

**Table 1.** Clinical Characteristics of Tubeless Insulin Management System Users by Age Group (Entire Cohort)

	Age category, year							
	0 to <5	5 to <10	10 to <15	15 to <20	20 to <30	30 to <40	>40	Overall
*n* (%)	77 (2.2)	532 (15.4)	1505 (39.8)	909 (25.2)	182 (4.6)	161 (4.5)	291 (8.4)	3657
Age, year	3.9 (3.2, 4.3)	7.8 (6.8, 8.9)	12.3 (11.0, 13.4)	16.4 (15.5, 17.5)	24.7 (22.1, 27.2)	34.2 (31.9, 37.1)	50.9 (46.3, 57.9)	13.7 (10.8, 17.3)
HbA1c, %^a^	7.2 (6.9, 8.2)	7.2 (6.7, 7.7)	7.5 (6.7, 7.7)	7.7 (7.0, 8.6)	7.6 (6.8, 8.5)	7.5 (6.8, 8.4)	7.6 (7.1, 8.3)	7.5 (6.9, 8.2)
Insulin dose, U/(kg·day)^a^	0.65 (0.47, 0.78)	0.66 (0.54, 0.77)	0.76 (0.61, 0.95)	0.80 (0.64, 1.0)	0.60 (0.42, 0.77)	0.47 (0.36, 0.60)	0.47 (0.37, 0.50)	0.71 (0.55, 0.90)
BMI-SDS^a^	0.96 (0.07, 1.53)	0.45 (−0.04, 0.92)	0.36 (−0.25, 1.07)	0.57 (−0.09, 1.20)	0.80 (0.24, 1.68)	0.79 (0.18, 1.69)	1.22 (0.48, 2.01)	0.55 (−0.09, 1.22)
DKA, %^b^	1.3	4.5	3.6	3.6	3.8	3.7	2.7	3.9
SH (coma), %^b^	2.6	0.7	1.6	1.8	0	1.8	2.0	1.5
SH (Level 3), %^b^	9.0	5.4	5.8	4.7	1.9	1.0	5.5	5.4
SMBG/day^a^	7 (5, 9)	6.5 (5, 8)	5 (4, 7)	5 (3.5, 6)	5 (3.5, 6)	4 (2, 6)	4 (4, 6)	5 (4, 7)
CGM (%)^a^	33.8	38.6	37.6	31.4	18.6	17.4	13.7	32.7
Previous therapy (tethered pump/MDI%)^a^	58/42	35/65	23/76	27/73	66/33	81/17	77/18	38/59
Retention rate (%)	100	96.4	94.8	83.3	94.0	95.7	96.6	92.4

Results are presented as median (IQR).

^a^ In a small number of patients from each age group, data were missing from each measure (up to 9% of patients for HbA1c, 11% for BMI-SDS, 16% for insulin dose, and 18% for SMBG). For better comparison, SDS-BMI was also calculated in the adult population using the 18-year old reference values.

BMI-SDS, body mass index-standard deviation score; CGM, continuous glucose monitor; DKA, diabetic ketoacidosis; HbA1c, hemoglobin A1c; IQR, interquartile range; MDI, multiple daily injections; SH, severe hypoglycemia.

### Glycemic control and frequency of acute complications for 3 years

The change in glycemic control and frequency of acute complications with long-term tubeless insulin pump use compared with prior treatment are summarized for patients with at least 1 year of data before switching to a tubeless insulin pump and at least 1 year of follow-up data (total cohort; *n* = 2911). In this population, there were *n* = 2873 diagnosed with type 1 diabetes, *n* = 10 with type 2 diabetes, and *n* = 28 with LADA/other. Fifty-eight percent had switched from MDI, 38% had switched from a tethered pump, and 3% started from onset with tubeless therapy, with children switching more frequently from MDI and adults more often from tethered pumps (Table 1). In the total cohort compared with the year before switch over the next 3 years, a continuous reduction of DKA was seen already in the first year (5.6% [(*n* = 2912] vs. 3.2% vs. 2.9% [*n* = 1921] vs. 2.2% [*n* = 1336])), and from year 2 a reduction in SH (level 3) (5.0% vs. 5.4% vs. 4.5% vs. 4.1%) and hypoglycemia with coma (1.3% vs. 1.4% vs. 0.9% vs. 0.6%) respectively, was seen, whereas Hba1c showed only a mild age-related increase from 7.5% to 7.7%. As the number of individuals with available data declined over the 3-year period, the analysis was repeated for the subgroup of patients (*n* = 1311) with data available for all 3 years of follow-up and a corresponding MDI cohort from the same centers (Table 2).

**Table 2.** Glycemic Control and Frequency of Acute Complications for 3 Years of Tubeless Insulin Pump Therapy in Patients with 3-Year Follow-Up Compared with Prior Treatment and with Multiple Daily Injection Patients with 3-Year Data from the Same Centers During the Same Time Period

	MDI^a^				Tubeless-pump^b^			
	Year prior				Year prior	Post-tubeless insulin pump		
Parameter	(*n* = 1874)	1 year (*n* = 1874)	2 years (*n* = 1874)	3 years (*n* = 1874)	(*n* = 1311)	1 year (*n* = 1311)	2 years (*n* = 1311)	3 years (*n* = 1311)
Age, year	12.3	13.4	14.4	15.4	11.5	12.4	13.5	14.5
Diabetes duration, year	3.4	4.4	5.4	6.4	3.3	4.2	5.3	6.2
HbA1c, %^c^	7.4	7.5	7.6	7.8	7.5	7.4	7.7	7.7
Insulin dose, U/(kg·day)^c^	0.83	0.87	0.92	0.95	0.77	0.74	0.77	0.79
BMI-SDS^c^	0.41	0.46	0.49	0.54	0.41	0.49	0.53	0.59
DKA, %^d^	3.1	3.0	3.4	3.3	6.3	3.7	3.1	2.2
SH (coma), %^d^	2.5	2.4	1.9	1.9	1.7	1.4	1.1	0.5
SH (level 3), %^d^	8.4	7.3	6.4	6.3	5.6	6.3	5.0	4.1
SMBG/day	6	6	5	5	6	6	5	5
CGM (%)	1.6	1.5	6.6	29.2	6.7	16.3	31.0	44.8

Data are shown as median or proportion.

^a^ Patients with type 1 diabetes on MDI in same centers with >10 tubeless pumps and 3 years follow-up, same treatment years. For the MDI group, the “Year Prior” corresponds to the third year before last year evaluated.

^b^ Patients who had at least 1 year of data before tubeless insulin pump initiation and 3 years of follow-up data available.

^c^ In a small number of patients, data were missing from each measure (total cohort: up to 7% for HbA1c, 8% for BMI-SDS, and 9% for insulin dose; subgroup with 3 years. follow-up: up to 4% for HbA1c, 5% for BMI-SDS, and 5% for insulin dose).

^d^ Patients with ≥1 event per year, %. SH, severe hypoglycemia.

**FIG. 1.**
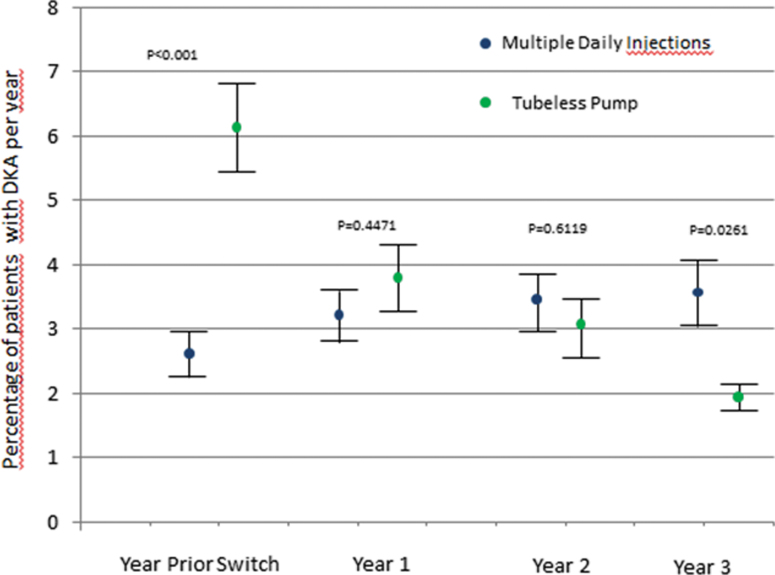
Data are shown for the cohort of 1311 patients with 3 years of follow-up data post-tubeless insulin pump initiation. Comparison of the estimated mean ± SEM of DKA (top panel) and SH (Level 3) (bottom panel) adjusted for age, diabetes duration, gender, baseline-HbA1c with either MDI (*n* = 1874) or tubeless pump (*n* = 1311) treated at the same centers during the same 3-year time period. For the MDI group, the “Year Prior” corresponds to the third year before last year evaluated. DKA, diabetic ketoacidosis; HbA1c, hemoglobin A1c; MDI, multiple daily injection.

Initially, CGM use was higher in the tubeless pump group, and after increasing over time in both cohorts, CGM was used approximately by one-third of the patients in either group (Table 2). The annual rate of DKA was 6.3% with prior treatment, and decreased to 3.7%, 3.1%, and 2.2% in years 1, 2, and 3, respectively, of tubeless insulin pump therapy (Fig. 1). The frequency of SH (coma) was 1.7% with prior treatment, and decreased after 1, 2, and 3 years of tubeless insulin pump therapy to 1.4%, 1.1%, and 0.5%, respectively. The rate of SH (Level 3) was 5.5% with prior treatment and increased slightly to 6.3% in the first year of tubeless insulin pump therapy. In year 2, the proportion of subjects with SH (Level 3) was lower than with prior treatment and continued to decrease in year 3 (5.0% and 4.1%, respectively).

The HbA1c was 7.5% with prior therapy, decreased slightly to 7.4% in the first year of tubeless insulin pump therapy, and increased to 7.7% in years 2 and 3. The decrease in the frequency of DKA, SH (coma), and SH (Level 3) ranged from 29% to 76% with tubeless insulin pump use compared with prior treatment in the total cohort and the subgroup with 3 years of follow-up data. The difference between tubeless pump and MDI remained significant after adjustment for age, diabetes duration, gender, and baseline HbA1c (Fig. 1) even after accounting for the differences in CGM use between pump and MDI (data not shown).

### Retention rate

Discontinuation of tubeless insulin pump treatment in patients with type 1 diabetes was 7.4% and occurred after 0.9 ± 1.2 year. The retention rate was well above 90% for all age groups, except for young adults aged 15 to 20 years (retention rate was >80% for this age group) (Table 1). The discontinuation rate in the total cohort of 2911 patients was similar (7.5%).

## Discussion

This retrospective analysis of the German/Austrian DPV registry is the first evaluation of acute complications with long-term tubeless insulin pump therapy, as well as the first characterization of tubeless insulin pump users of all ages outside of the United States. This analysis demonstrated that tubeless insulin pump therapy is associated with a low rate of SH and DKA in a primarily pediatric and adolescent population that is prone to these acute complications. The frequency of DKA and SH decreased after 3 years of tubeless insulin pump use compared with prior treatment modality and compares favorably with previous reports of youth using traditional insulin pumps from this and other large registries.^3–8,27^

Much of the decline of DKA was driven by the significantly higher rate at baseline compared with MDI. Potentially DKA episodes have contributed to the decision to switch the therapeutic regimen and/or led to additional measures. Nevertheless, over time the frequency of DKA and SH declines progressively after the switch and was found to be significantly lower than in a contemporary MDI group from the same centers after adjustment for common variables of influence such as age, diabetes duration, gender, HbA1c, or CGM use. Of course, this statistical difference may still be an artifact due to the limitations of real-world data and would need confirmation in a proper randomized controlled trial. Although the frequency of acute complications decreased in the 3 years after initiation of tubeless insulin pump therapy, glycemic control as measured by HbA1c remained consistent with or better than overall population data from this and other large registries.^3,21,35,36^ It is interesting that the HbA1c for people of ages from 15 to 30 years is higher than for other age groups, pointing to the challenges of the transition period from pediatric to adult care.

Glycemic control improved in the first year of tubeless insulin pump therapy, but increased moderately in years 2 and 3. Improvements in glycemic control with use of the tubeless pump in the first year were also reported in a previous analysis of the DPV registry^30^ and elsewhere.^37^ The trend toward an increased HbA1c beyond the first year may be explained by the expected age-related worsening in glycemic control associated with adolescents going through puberty, which is consistent with observations made previously both with tubeless^30^ and tethered pumps.^38^

In this retrospective analysis, high treatment retention rates (>90%) were observed in patients with type 1 diabetes of most age groups after 1 year of tubeless insulin pump use; young adults aged 15 to 20 years had a retention rate >80%. The high treatment retention is comparable with other real-world studies in pediatric and adult tubeless pump users.^39,40^

Adolescents have been reported to have the highest proportion of subjects with DKA, between 5.6% and 8.4% in the 13- to 17-year age group,^4^ and thus the comparatively low DKA frequency of 2.2% observed after 3 years of tubeless insulin pump therapy in this study is of clinical interest. Many younger tubeless insulin pump users had switched from MDI treatment, whereas in the age group >30 years, the majority of patients switched from a pump with catheter to a tubeless pump. This may account, in part, for the low proportion of subjects with DKA that is consistent with other reports, indicating that MDI use is associated with significantly higher rates of DKA in youth compared with traditional insulin pump use.^3,6,21,25^ However, the risk of DKA is still of concern as interruptions in insulin delivery and infusion site issues can quickly cause hyperglycemia, which can progress to DKA if untreated.^25,33,41,42^

Although several aspects of insulin pump therapy may contribute to improved glycemic outcomes, one may argue that the required pump site change at 72 h with this tubeless insulin pump system may potentially alleviate some of the infusion site issues that contribute to hyperglycemia and the increased potential for DKA.^40,43^ Of note, the frequency of DKA of 2.2% in this study compares favorably with the frequency of DKA reported for insulin pump users overall from the U.S. T1D Exchange (T1DX) registry (5.2% [4] and the German/Austrian DPV registry (3.4% [(3] to 5.2%).^4^

The proportion of subjects with SH (coma) was low initially but continued to decrease each year for 3 years' tubeless insulin pump use compared with prior treatment. The general limitation to real-world data is lacking information on the ascertainment rate that may underestimate the prevalence of SH. This finding of low SH (coma) is also consistent with previous research in which a significantly lower frequency of SH with insulin pump use has been observed compared with MDI.^3,6,21^ The frequency of 0.5% SH (coma) is lower than that reported for youth overall in the U.S. T1DX registry (4.9%)^5^ and for youth using insulin pumps in the German/Austrian DPV registry (1.8%).^3^ The decrease in SH (coma) observed with tubeless insulin pump use is important, as it may be associated with a commensurate decrease in hospital admissions and cost per year, as well as a decreased risk of mortality.^44–46^

Although the proportion of subjects with SH (Level 3) increased slightly during the first year of tubeless insulin pump therapy, in year 2, it was lower than the year before tubeless insulin pump initiation and continued to decrease further over time. In comparison, a recent study by Karges and colleagues of youth in the German/Austrian DPV registry found SH (Level 3) frequency to be 7.3% in MDI users and 5.5% in pump users, with a significantly higher rate of events per 100 patient-years in MDI users (*P* < 0.001).^3^ It is possible that SH is not frequently reported due to the inconsistency with which patients may interpret the definition of requiring assistance in the context of their experiences that could add variability to the results, potentially leading to a higher margin of error associated with this outcome.^7,8^ Nevertheless, both the improvements in DKA and hypoglycemia compare favorably with the contemporary MDI group in these centers.

Key strengths of this study include the large sample size, long-term follow-up, and the robust nature of the German/Austrian DPV registry. Limitations of the study are inherent in a retrospective design. In contrast to controlled trials, such real-world data on declining frequencies of acute complications associated with tubeless pumps preclude conclusions on causality. A direct comparison of these groups is compromised as no data is available on the clinical decision making for choosing MDI or tubeless pumps. Potential influencing factors such as educational status of the patient and/or their parents, training on nutrition, psychological support, or other additional care related to diabetes are not captured by the DPV registry.

At the time of the analysis, the only available tubeless pump in the DPV registry was the Omnipod. Possibly, similar associations could be seen with tethered pumps or other brands of tubeless pumps that are not currently available in Germany or Austria. In addition, other treatment changes made during the time period studied may have affected the results, including the adoption or discontinuation of a CGM^34,35,47^ or adjustments to pump therapy parameters. The principles of DPV preclude analyses that directly compare single commercial entities with each other. Thus, no comparative analysis was done between different brands of pumps or tubeless and tethered pumps. The results of the study may be influenced by the inclusion of some adults >20 years of age in the 3-year outcome data; however, the median age is indicative of a primarily pediatric/adolescent population. Compared with other large cohort studies, the study population was in fairly good glycemic control that may limit the generalizability of our findings.

## Conclusions

This large retrospective analysis of the German/Austrian DPV registry demonstrated that tubeless insulin pump therapy is associated with a low frequency of SH and DKA in a primarily pediatric and adolescent population that is prone to these acute complications. Despite the typical age-dependent increase in HbA1c through adolescence, glycemic control with tubeless insulin pump use compares favorably with other large registry data.^21,34^ In addition, high treatment retention rates were observed in patients with type 1 diabetes of all ages initiating tubeless insulin pump use.
